# Uniparental Genome Elimination in Australian Carp Gudgeons

**DOI:** 10.1093/gbe/evab030

**Published:** 2021-02-16

**Authors:** Zuzana Majtánová, Dmitrij Dedukh, Lukáš Choleva, Mark Adams, Petr Ráb, Peter J Unmack, Tariq Ezaz

**Affiliations:** 1 Laboratory of Fish Genetics, Institute of Animal Physiology and Genetics, Czech Academy of Sciences, Liběchov, Czech Republic; 2 Department of Biology and Ecology, Faculty of Science, University of Ostrava, Ostrava, Czech Republic; 3 Evolutionary Biology Unit, South Australian Museum, North Terrace, Adelaide, SA, Australia; 4 School of Biological Sciences, The University of Adelaide, SA, Australia; 5 Centre for Applied Water Science, Institute for Applied Ecology, University of Canberra, ACT, Australia; 6 Centre for Conservation Ecology and Genetics, Institute for Applied Ecology, University of Canberra, ACT, Australia

**Keywords:** genome elimination, hemiclone, hybridogenesis, unisexual, gametogenesis, *Hypseleotris*

## Abstract

Metazoans usually reproduce sexually, blending the unique identity of parental genomes for the next generation through functional crossing-over and recombination in meiosis. However, some metazoan lineages have evolved reproductive systems where offspring are either full (clonal) or partial (hemiclonal) genetic replicas. In the latter group, the process of uniparental genome elimination selectively eliminates either the maternal or paternal genome from germ cells, and only one parental genome is selected for transmission. Although fairly common in plants, hybridogenesis (i.e., clonal haploidization via chromosome elimination) remains a poorly understood process in animals. Here, we explore the proximal cytogenomic mechanisms of somatic and germ cell chromosomes in sexual and hybrid genotypes of Australian carp gudgeons (*Hypseleotris*) by tracing the fate of each set during mitosis (in somatic tissues) and meiosis (in gonads). Our comparative study of diploid hybrid and sexual individuals revealed visually functional gonads in male and female hybrid genotypes and generally high karyotype variability, although the number of chromosome arms remains constant. Our results delivered direct evidence for classic hybridogenesis as a reproductive mode in carp gudgeons. Two parental sets with integral structure in the hybrid soma (the F1 constitution) contrasted with uniparental chromosomal inheritance detected in gonads. The inheritance mode happens through premeiotic genome duplication of the parental genome to be transmitted, whereas the second parental genome is likely gradually eliminated already in juvenile individuals. The role of metacentric chromosomes in hybrid evolution is also discussed.

Significance

Most animals reproduce sexually, via biparental fusion of gametes resulting from the well-studied process of meiosis. However, only little is known about meiotic behavior of chromosomes and the extent of genetic recombination in animal groups that practice “asexual” reproduction. Here, we demonstrate that male and female hybrids of Australian carp gudgeons (*Hypseleotris*) are fertile and display uniparental chromosomal elimination, without genetic recombination, both as predicted by nuclear DNA markers. We show that genome elimination occurs premeiotically in juveniles, prior to gonad maturation. Asexual vertebrates are important animal models in research fields as diverse as speciation, toxicology, and unorthodox reproduction, for example, understanding how vertebrates clone their germ cells. These findings further enhance the potential of carp gudgeons in this regard.

## Introduction

The great majority of metazoan organisms reproduce through sex in a two-stage process, commencing with meiosis in each parent and culminating in the fusion of one ploidy-reduced, genetically shuffled gamete from each parent into a single zygote (Crow and Kimura 1965; Kondrashov 1988). In sexual reproduction, both paternal and maternal genomes enter meiosis. In meiosis I, the parental chromosomes pair, and genetic material undergo regular DNA-recombination. The paired chromosomes are then segregated to the opposite poles of the meiotic spindle, whereas in meiosis II, sister chromatids disjoin (Petronczki et al. 2003; Suwa and Yamashita 2007). The resulting haploid gametes contain a unique cocktail of recombinant chromosomal DNA from the mother and father. In this way reproducing animals we call “sexual.” Although the meiotic molecular machinery is highly conserved among Metazoans (Bernstein and Bernstein 2010), gametogenesis has been repeatedly modified in a small proportion of organisms, giving rise to clonal, or asexual, taxa.

To transfer a whole intact genome from multicellular animal to next generation in a gamete, those taxa exploit a wide range of cytogenetic mechanisms ranging from processes completely without meiosis (apomixis) to those with modified meiosis (automixis) (Stenberg and Saura 2009; Lenormand et al. 2016). Hybridogenesis is a reproduction mode unique in its ability to transmit not a whole, but one-half of a somatic genome via genome elimination. The process of uniparental genome elimination selectively eliminates either the maternal or paternal genome from germ cells (Gardner and Ross 2014; Dedukh, Riumin, et al. 2020). During uniparental genome elimination, only one parental genome is thus selected for transmission; it then undergoes chromosome doubling and enters meiosis (Tunner and Heppich 1981; Heppich et al. 1982; Dedukh et al. 2015). In contrast to standard Mendelian inheritance, the resulting haploid gametes are clones (in the absence of mutation) of the gametes produced in the preceding generation (Doležálková-Kaštánková et al. 2018). Haploidization via chromosome elimination is a fairly common phenomenon in plants, with basic research leading to applications in accelerated plant breeding (Sanei et al. 2011; Comai 2014; Ishii et al. 2016). However, it remains a poorly understood process in animal ontogenesis.

Classic hybridogenesis, commonly also referred to as hemiclonal inheritance, has been demonstrated in a small range of organisms from various taxonomic groups, such as fishes from the genera *Poeciliopsis* (Schultz 1961), *Squalius* (Carmona et al. 1997), or *Hexagrammos* (Kimura‐Kawaguchi et al. 2014; Munehara et al. 2016), frogs from the *Pelophylax esculentus* complex (Tunner 2009), and stick insects in the genus *Bacillus* (Mantovani and Scali 1992). In addition, it has also been inferred in several other groups, most notably *Hypseleotris* fishes (Bertozzi et al. 2000; Schmidt et al. 2011) but full confirmation ideally relies upon detailed chromosomal investigation.

Advancements in molecular cytogenomics offer a spectrum of molecular tools allowing for new empirical insights into uniparental genome elimination. Research in various animal models has identified that DNA elimination mostly occurs before the initiation of meiosis (Cimino 1972; Ogielska 1994; Scali et al. 2003; Schön et al. 2009; Stenberg and Saura 2009; Dedukh et al. 2015, 2017; Dedukh, Riumin, et al. 2020). As shown in *Pelophylax* water frogs, DNA elimination seems to be a gradual process of individual chromosomes enclosed within micronuclei (Chmielewska et al. 2018; Dedukh, Riumin, et al. 2020). To allow the noneliminated chromosome set (the one to be transmitted) to enter effectively into meiosis, doubling of a haploid parental chromosome set must occur through 1) premeiotic genome endoreplication (cell cycle without mitosis), or 2) endomitosis (mitosis without chromosome segregation) (Dedukh, Riumin, et al. 2020). However, studies of insects and triploid vertebrates have shown that elimination of the uniparental chromosomal set may take place during meiosis as well (Zhang et al. 1998; Nabais et al. 2012; Gardner and Ross 2014). The inclusion of a wider concept of hybridogenesis into uniparental genome transmissions extends the number of mechanistic processes, from which genome elimination may be absent (Doležálková et al. 2016; Lavanchy and Schwander 2019). Therefore, to understand these processes, comprehensive and case-specific studies are needed to demonstrate any discordance in genomic content between a zygote, soma, and germ cells, and to identify pathways of elimination and transmission of genomes.

Carp gudgeons (*Hypseleotris*, Eleotridae) are a genus of small fishes with a widespread distribution across the Indo-Pacific, including moderate diversification in Australian freshwater environments (Thacker and Unmack 2005). Eastern Australia contains a species complex consisting of two described species (*Hypseleotris klunzingeri* and *Hypseleotris galii*), and several undescribed species (Hoese et al. 1980; Unmack 2000). In addition to four sexually reproducing species, Bertozzi et al. (2000) described the co-occurrence of three apparent F_1_ hybrid forms derived from three distinctive taxa revealed in the lower Murray River and first suggested these were unisexual carp gudgeons. Subsequently, Schmidt et al. (2011) used microsatellite markers and proposed the occurrence of hybridogenesis maintaining the coexistence of male and female hybrid lineages and sexual species. More recently, Unmack et al. (2019) discovered one of the missing sexual parental species using SNPs. However, despite these population analyses and the demonstrated hemiclonal nature of several lineages (Unmack et al. 2019), it remains unclear what enables the persistence of this reproductive system from a cellular perspective.

In this article, we explore the proximal cytogenomic mechanisms maintaining the carp gudgeon’s hybrid genotypes as F_1_’s. We analyzed somatic and germ cell chromosomes, as well as gonadal microanatomy in both hybrid and sexual individuals. Using genomic in situ hybridization (GISH) with species-specific probes, we identified parental chromosomal sets in various hybrids and traced the destiny of each set during mitosis (in somatic tissues) and meiosis (in gonads). We specifically tested whether 1) chromosomal behavior supports a hypothesis of uniparental genome elimination and whether 2) reproduction mode of hybrids is linked with cytological principles of classic hybridogenesis.

## Materials and Methods

### Study Species

For a clarity, here we use the term “sexual,” or “sexual species,” for taxa of male and female individuals that use the regular meiotic (sexual) cycle. We also use the term “hybrid” for male and/or female individuals having parental chromosomal sets in their soma from the extant sexual species, and displaying uniparental chromosomal elimination in their reproduction cycle. Five sexual species have been recognized in eastern Australia: two, *Hypseleotris compressa* and *H. klunzingeri* frequently co-occur with the sexual/unisexual complex, but there is no record of them being involved in any hybridization. The sexual/unisexual complex consists of three sexual species, *H. galii*, *H.* sp. Midgley’s, and *H.* sp. Bald, which have traditionally been identified in previous papers (Bertozzi et al. 2000; Schmidt et al. 2011; Unmack et al. 2019) by the codes HA, HB, and HX, respectively, which we use from this point forward in this article. Each of the three known interspecific diploid hybrid genotypes have the F_1_ genomic combinations designated as HA×HB, HA×HX, and HB×HX. With the exception of HX, which has an extremely restricted distribution (Unmack et al. 2019), the other species and hybrids in the sexual/unisexual complex are widespread across the Murray–Darling Basin (1,059,000 km^2^), in addition HB and HB×HX are present in the Bulloo River (75,610 km^2^) and Cooper Creek catchments (298,000 km^2^) and HA×HB is present in coastal rivers from at least the Clarence River north to Waterpark Creek (110,000 km^2^). There is a strong sex bias in some hybrids, with HA×HB being strongly male biased (Schmidt et al. 2011 recorded 80 males, 2 females plus 5 indeterminate), HA×HX was strongly female biased (Schmidt et al. 2011 recorded 7 males, 152 females, and 9 indeterminate). While the sex ratio varies, HB×HX typically has both sexes present at most sites where they are found (Unmack et al. unpublished data).

### Studied Material

We examined 33 individuals from 10 localities across eastern Australia (supplementary table S1, Supplementary Material online). We analyzed representatives of four sexual species: HA, HB, HX, and *H. klunzingeri* (HK) which was included as an outgroup as it is the sister species to the sexual/unisexual complex (Unmack et al. 2019). Two types of putative hybridogenetic hybrids were examined: HA×HB and HB×HX. A subset of 17 individuals was used for karyotype analyses and identification of somatic versus gonadal differences. The gonadal structure of 16 individuals was examined through confocal microscopy. All wild samples were obtained under state fisheries permits, and research was conducted with approval from the University of Canberra Ethics Committee (CEAE.15-05). Each individual was anesthetized with an overdose of clove oil. Complete information about the number of individuals, sex, localities, and methods employed is provided in supplementary table S1, Supplementary Material online.

### Genotype Identification—DArT Sequencing

Genotypes of most individuals were confirmed via SNPs generated using DArTseq (DArT Pty Ltd.), a variation of the double-digest RAD technique as described by Kilian et al. (2012). The R-package dartR 1.8.3 (Gruber et al. 2018) was used for filtering the data, generating PCA plots and for exporting data for phylogenetic analysis to enable species identification. More information is provided in Supplementary Methods.

### DNA Flow Cytometry

The genome size of cell populations from the testes was estimated by measurement of the cell nuclei using a BD FACSAria II flow cytometer on a subset of 17 individuals (same individuals as used for karyotype analyses; table 1 and supplementary table S1, Supplementary Material online). Testes and muscle tissues were fixed in 70% ethanol prior to measurements. Testes and muscle tissues were minced in 0.1% Triton X100, 10 µ/ml DAPI and 15 mM MgCl_2_ to release nuclei from cells. These nuclei suspensions were incubated at +4 °C overnight. After incubation of nuclei suspensions for 4–6 h (at +4 °C), they were analyzed by BD FACSAria flow cytometer. At least 10,000 events were measured. BD FACSDiva software (6.1.3) was used to process the obtained data. Suspension of nuclei released from muscle cells was used as an internal control.

**Table 1:** Chromosomal Characteristics of Individuals Under the Study

#	ID	Genotype	Locality	2*n* in Somatic Cells			2*n* in Germ Cells			Genome Sets Distinguished Based on GISH		
				2*n*	m/sm	st/a	2*n*	m/sm	st/a	Somatic Cells		Germ Cells
1.	HA×HB_1	HA×HB	Angas	46	2	44	44	4	40	**B genome = 2 m/sm + 20st/a**	A genome** = **24 st/a	BB genome** = **4 m/sm** + **40st/a
2.	HA×HB_2	HA×HB	Angas	46	2	44	44	4	40	**B genome = 2 m/sm + 20st/a**	A genome** = **24 st/a	BB genome** = **4 m/sm** + **40st/a
3.	HA×HB_3	HA×HB	Angas	46	2	44	44	4	40	**B genome = 2 m/sm + 20st/a**	A genome** = **24 st/a	BB genome** = **4 m/sm** + **40st/a
4.	HA×HB_4	HA×HB	Angas	46	2	44	44	4	40	**B genome = 2 m/sm + 20st/a**	A genome** = **24 st/a	BB genome** = **4 m/sm** + **40st/a
5.	HA×HB_5	HA×HB	Mudgeeraba	45	3	42	42	6	36	**B genome = 3 m/sm + 18st/a**	A genome** = **24 st/a	BB genome** = **6 m/sm** + **36st/a
6.	HA×HB_6	HA×HB	Byfield	47	1	46	46	2	44	**B genome = 1 m/sm + 22st/a**	A genome** = **24 a	BB genome** = **2 m/sm** + **44st/a
7.	HB×HX_1	HB×HX	Gwydir	47	1	46	46	2	44	B genome** = **24st/a	**X genome = 1 m/sm + 22a**	XX genome** = **2 m/sm** + **44st/a
8.	HB×HX_2	HB×HX	Faithful	48	0	48	48	0	48	B genome** = **24st/a	X genome** = **24 st/a	XX genome** = **48st/a
9.	HB×HX_3	HB×HX	Faithful	48	0	48	48	0	48	B genome** = **24st/a	X genome** = **24 st/a	XX genome** = **48st/a
10.	HB×HX_4	HB×HX	Gwydir	46	2	44	46	2	44	B genome** = **1 m/sm** + **22st/a	X = 1 m/sm** + **22st/a	XX genome** = **2 m/sm** + **44st/a
11.	HB_1	HB	Gwydir	47	1	46	47	1	46	No specif. signal	No specif. signal	No specif. signal
12.	HB_2	HB	Gwydir	48	0	48	48	0	48	—	—	—
13.	HA_1	HA	Byfield	48	0	48	48	0	48	—	—	—
14.	HK_1	HK	Yabba	48	0	48	48	0	48	—	—	—
15.	HK_2	HK	Yabba	48	0	48	48	0	48	—	—	—
16.	HK_3	HK	Yabba	48	0	48	48	0	48	—	—	—
17.	HX_1	HX	Urumwalla	48	0	48	48	0	48	—	—	—

Notes: Codes refer to sexual species, *H. galii* (HA), *H*. sp. Midgley’s (HB), *H*. sp. Bald (HX) and *H. klunzingeri* (HK) and interspecific F_1_ hybrid genotypes designated as HA×HB and HB×HX; B genome, haploid genome of HB; A genome, haploid genome of HA; X genome, haploid genome of HX; m/sm, meta-submetacentric; st/a, subtelocentric–acrocentric chromosomes; transmitted genomes are highlighted with bold font.

### Chromosome Preparation

Metaphase chromosomes were prepared according to Bertollo et al. (2015) with slight modifications. Briefly, fish were injected with 0.1% colchicine solution (1 ml/100 g of body weight) 45 min before being sacrificed using an overdose of anesthetic. The kidneys, gills, part of spleen, and guts were dissected in 0.075 M KCl at room temperature. The cell suspension free of tissue fragments was hypotonized for 30 min in 0.075 M KCl, fixed in freshly prepared fixative (methanol:acetic acid 3:1, v/v), washed twice in fixative and spread onto microscope slides. For inspection of chromosomal composition and structure in germ cells metaphases and germ cells meiotic metaphases I, suspensions from testes were prepared, using the same protocol, with hypotonization prolonged to 45 min. The same protocol cannot be used for females as they have a low number of dividing cells as well as large yolky oocytes, preventing examination of meiosis using classical cytogenetic methods.

### Cytogenetic Analyses

Mitotic metaphase chromosomal preparations from all individuals were stained with 5% Giemsa solution for 10 min to confirm ploidy and morphology of chromosomes. To confirm the genome composition in hybrid individuals and to detect possible genome elimination, GISH was performed on chromosomes obtained from both somatic and gonadal tissue of 12 putative hybridogenetic hybrids. Probes used in GISH experiments were prepared from whole genomic DNA (gDNA) of the three parental sexual HA, HB, and HX. gDNA was extracted from muscles using the DNeasy Blood and Tissue Kit (Qiagen, Hilden, Germany) according to the manufacturer’s instructions. gDNA samples were labeled via nick translation using Fluorescein Nick Translation Labeling Kit (Jena Bioscience, Jena, Germany) and Cy3 Nick Translation Labeling Kit (Jena Bioscience) following the protocol supplied by the manufacturer. The best results were obtained after 45 min of nick translation until labeled DNA fragments were 200–500 bp long. Species-specific hybridization probes combined gDNA of sexual species (HB with either HA or HX) to perform GISH experiments on chromosomes of hybrid individuals (table 1). Salmon sperm was used as a blocking reagent for repetitive DNA. The hybridization and detection procedure were carried out under conditions described by Majtánová et al. (2016). The chromosomes were counterstained with Vectashield/DAPI (1.5 mg/ml) (Vector, Burlingame, CA).

### Microscopy and Image Analyses

Chromosomal preparations were examined by a Zeiss Axioplan epifluorescence microscope equipped with a CCD camera and a ZEISS Axio Imager.Z2 epifluorescence microscope (Zeiss, Oberkochen, Germany). Images of metaphase chromosomes were recorded with a CoolCube 1 camera (MetaSystems, Altlussheim, Germany). The IKAROS and ISIS imaging programs (Metasystems) were used to analyses gray‐scale images. The captured digital images from GISH experiments were pseudocolored (red for Anti‐Digoxigenin‐Rhodamin, green for Invitrogen FITC‐Streptavidin) and superimposed using Adobe Photoshop software (CS5).

#### 3D Immunofluorescence Staining

To compare gonadal morphology between sexual and hybrid individuals as well as to identify the ontogenetic stage when genome elimination occurs, we examined gonads of 16 individuals (supplementary table S1, Supplementary Material online) under a laser scanning confocal microscope. Juveniles (1–2 months, 10 mm long) and adult fish were sacrificed using an anesthetic overdose. The caudal muscle tissue of each individual was collected in 80% ethanol for genotyping using DArT sequencing. The main body (for juveniles) or part of gonads (in case of adults) was fixed in 2% paraformaldehyde for 10 h and then transferred to 1× PBS with 0.02% NaN_3_ for long term storage. Prior to immunofluorescence staining, gonads were placed in 1% solution of Triton X100 in 1× PBS and incubated for 4–5 h at room temperature. Afterward, tissues were washed in 1× PBS at room temperature and incubated for 1–2 h in a 1% blocking solution (Roche) prepared with 1× PBS. Germ cells were visualized by rabbit polyclonal antibodies against Vasa protein (DDX4 antibody [C1C3], GeneTex, Irvine, CA). Incubation with primary antibodies was carried out at room temperature overnight, followed by washing in 1× PBS with 0.01% Tween (ICN Biomedical Inc.). Secondary antibodies conjugated with Alexa-594-conjugated goat anti-rabbit IgG (H + L) (Thermo Fisher Scientific, Waltham, MA) were added according to the manufacturer's instructions and incubated for 12 h at room temperature. Tissues were then washed in 1× PBS with 0.01% Tween (ICN Biomedical Inc.) and counterstained with DAPI (1 µg/µl) (Sigma) in 1× PBS at room temperature for overnight.

### Confocal Laser Scanning Microscopy

Tissues were placed in a drop of DAPI with Vectashield (1.5 mg/ml) (Vector, Burlingame, CA), solution and mounted with cover slides and examined under Leica TCS SP5 confocal microscope based on the inverted microscope Leica DMI 6000 CS (Leica Microsystems, Germany). Specimens were analyzed using HC PL APO 20×, 40×, and 63× objective. Diode and helium-neon lasers were used to excite the fluorescent dyes DAPI and fluorochrome Alexa-594, respectively. The images were captured using LAS AF and processed in LAS AF Lite software (Leica Microsystems, Germany).

## Results

### Genotype Identification—DArT Sequencing

All results obtained from DArT sequencing are provided in the Supplementary Materials. Genotyping confirmed the identifications of all individuals examined in this study as provided in table 1, supplementary table S1 and supplementary figures S2–S5, Supplementary Material online. We were unable to sequence juvenile individuals from Albury and Wagga Wagga, but these populations only consisted of HB and HB×HX adults at the time when the juveniles were collected.

### Karyotype Variability among Analyzed Individuals

We observed variability in numbers and morphology of chromosomes obtained from somatic tissue among the studied individuals. Whereas some individuals displayed a karyotype 2*n* = 48, composed of solely acrocentric chromosomes, others exhibited chromosomal counts ranging from 45 to 47 with one to three metacentric chromosomes. All individuals, regardless of the chromosomal number, displayed constant chromosome arm numbers, NF = 48. Detailed information about karyotype composition for each individual is provided in table 1.

### Karyotype Differences of Mitotic Spreads between Somatic and Gonadal Tissues in Hybrids

**Fig. 1.**
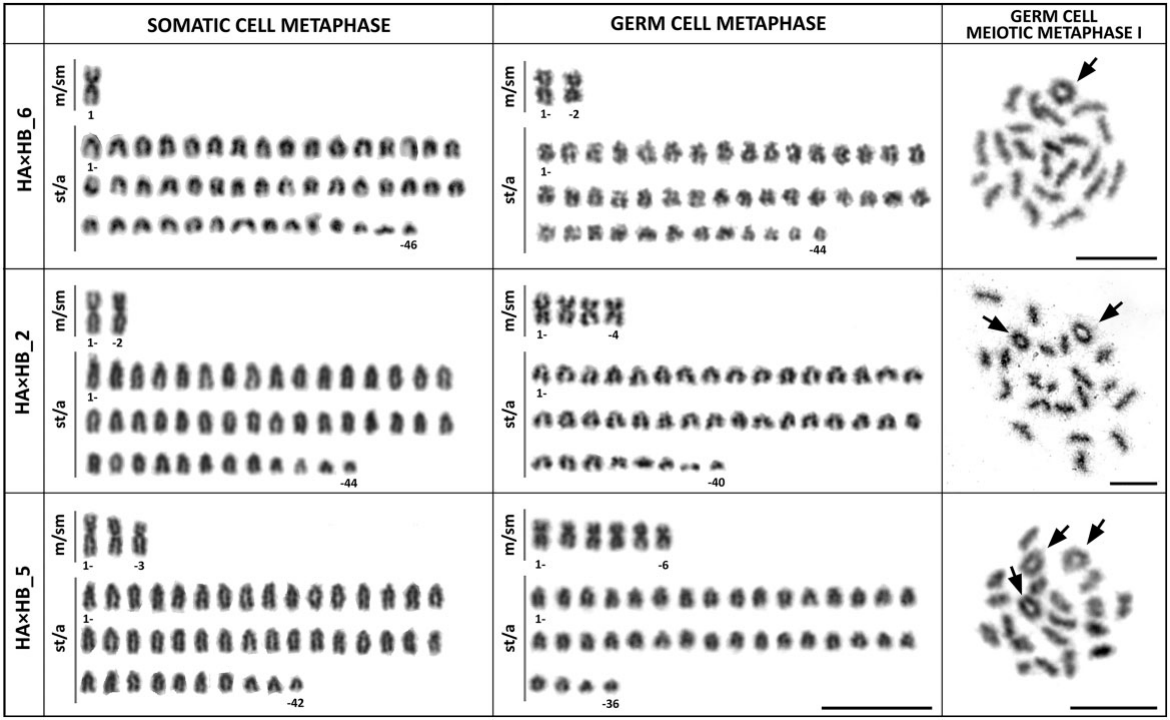
Karyotype differences between somatic cells and germ cells in hybrid individuals HA×HB (*Hypseleotris galii* × *H.* sp. Midgley’s). Giemsa-stained karyotypes obtained from somatic cells (first column), germ cells (second and third column). m/sm, meta-submetacentric; st/a, subtelocentric–acrocentric chromosomes. In meiotic metaphase I, we observed bivalents forming circles (arrows). The numbers of such bivalents correspond to pairs of metacentric chromosomes observed in mitosis of germ cells after the expected elimination of one genome and duplication of the second one (i.e., hybridogenetic reproduction). Bars equal 10 µm.

In hybrid individuals, we observed within-individual differences in chromosome classification and numbers. These characters can be used to compare metaphases obtained from somatic tissues (mixed kidneys, gills, and livers) and gonadal tissue (testes) (fig. 1), because the absence of chromosomes characterizing a specific chromosomal set in one tissue may indicate their programmed loss. Differences in the chromosome numbers suggest the possible premeiotic elimination of one genome and subsequent duplication of the other in these individuals (fig. 1). Detailed information about differences of chromosome numbers between somatic and germ cells for each individual are provided in table 1. In addition to the mitotic metaphase spreads obtained from gonads, we observed also other stages of meiosis, that is, metaphase I. These spreads consist of bivalents. The numbers of such bivalents correspond to pairs of chromosomes observed in mitosis of germ cells after the expected elimination of one genome and duplication of the second one (i.e., hybridogenetic reproduction). The pairs of metacentric chromosomes (if presented), formed circle-shaped bivalent (fig. 1).

### Chromosomal Evidence of Hybridogenesis via Genome Elimination

**Fig. 2.**
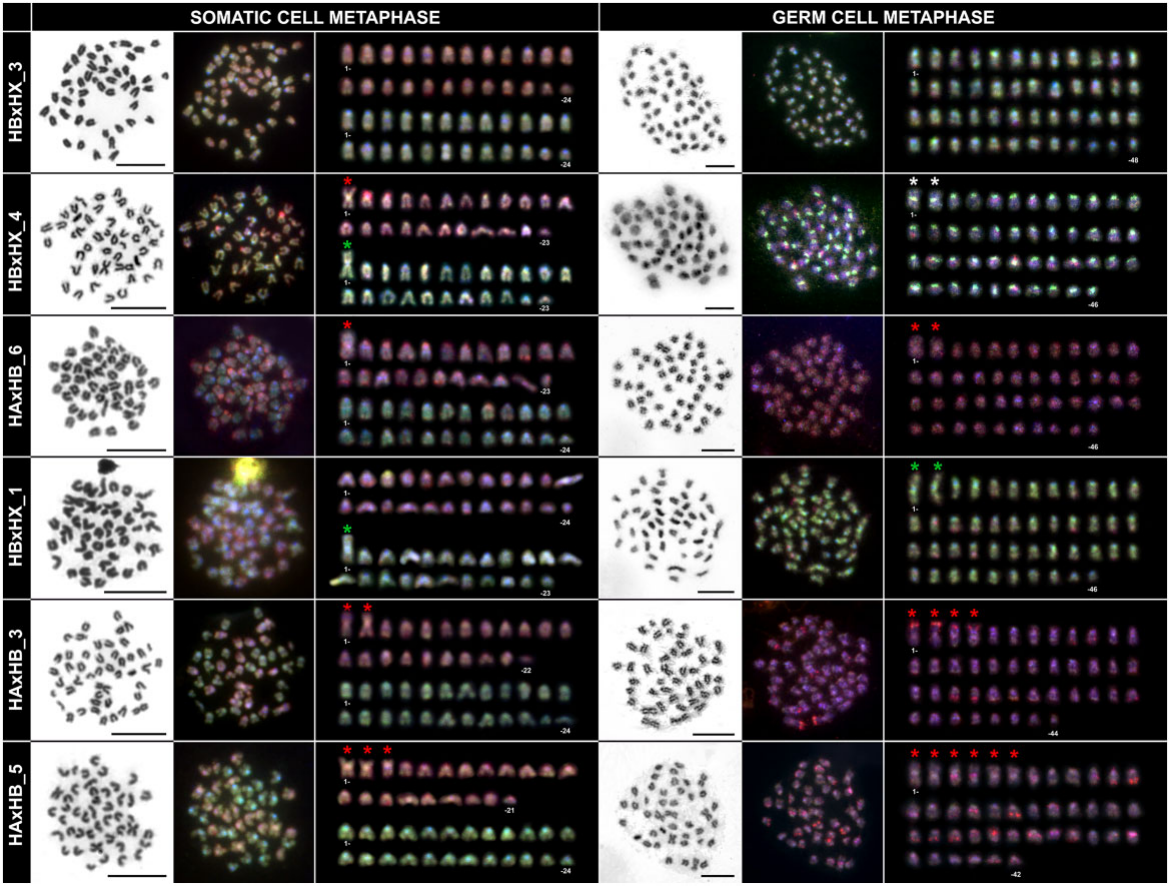
Genomic in situ hybridization (GISH) in somatic and germ cells of hybrid individuals. Both haploid parental chromosomal sets were clearly distinguishable in metaphases obtained from somatic tissue after GISH. In metaphases obtained from gonadal tissue, only one parental genome was detected. Red dye represents *H.* sp. Midgley’s (HB) gDNA; green dye represents *Hypseleotris galii* (HA) gDNA or *H.* sp. Bald (HX) gDNA. To visualize the proper morphology of chromosomes, Giemsa stained metaphase spreads are presented. Chromosomes are arranged in a decreasing size order, metacentric/submetacentric chromosomes are marked with asterisks. Bars equal 10 µm.

GISH was performed on 12 hybrid and one sexual individual used as a control (table 1 and supplementary table S1, Supplementary Material online) to identify parental chromosomal sets. Both haploid parental chromosomal sets were clearly distinguishable in metaphases obtained from somatic tissue in all hybrids (fig. 2, left panel). The metaphase chromosomes showed no visible intergenomic exchanges between the parental sets, supporting a scenario of the genomic integrity and F_1_ hybrid state on a whole-chromosomal level. In metaphases obtained from gonadal tissue (germ cells), only one parental genome was detected after GISH staining (fig. 2, right panel). Based on the identification of haploid parental chromosomal sets in somatic cells, we were able to distinguish which genome is presented in gonads. In all HA×HB hybrids, we only detected the parental HB genome (corresponding to parental species *H*. sp. Midgley’s) in gonadal metaphases (fig. 2, table 1). That means that parental genome HA (corresponding to *H. galii*) was eliminated, followed by duplication of HB genome (table 1). Based on these observations we conclude that such individuals uniparentally transmit only the HB genome into their gametes. In HB×HX hybrids, we observed that parental HB genome was eliminated. Nevertheless, in two cases (i.e., IDs: HB×HX_3 and HB×HX_4; fig. 2), when the parental haploid sets consist of the morphologically same karyotypes we cannot clearly conclude which genome is propagated without using more specific cytogenetic markers.

### Gonadal Structure of Hybrid and Sexual Individuals

As hybrid males are absent or rare in most sexual/unisexual complexes, we analyzed their ability to produce sperm via DNA flow cytometry. We analyzed seven sexual and 10 hybrid adult males (supplementary table S1, Supplementary Material online). Both hybrid and sexual individuals possessed haploid (1C, corresponding to spermatids and spermatozoa), diploid (2C, corresponding to spermatogonia and somatic cells) and cells after DNA synthesis cell populations (4C, corresponding to primary spermatocytes) (supplementary fig. S1, Supplementary Material online). We also did not observe the accumulation of aneuploid cells (supplementary fig. S1, Supplementary Material online). Our results suggest that meiosis in hybrids likely does not affect regular spermatogenesis and that hybrid males are able to produce haploid sperm.

**Fig. 3.**
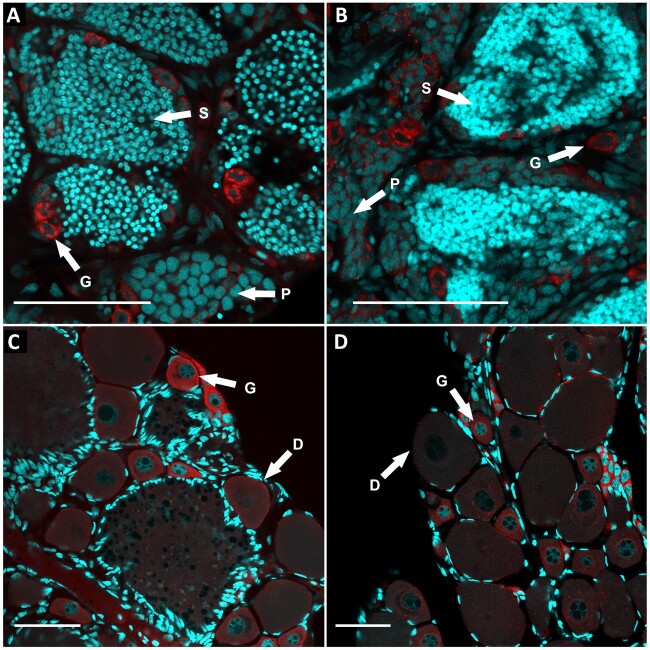
Comparison of gonadal microanatomy in sexual and hybrid individuals. Whole-mount immunofluorescent staining with antibodies against Vasa protein (red) identifying germ cells (G). DAPI is visualizing chromatin (cyan). (*A*) Sexual male *Hypseleotris klunzingeri* (ID: HK_3); (*B*) hybrid male HB×HX (*H.* sp. Midgley’s × *H.* sp; ID: HB×HX_7); (*C*) sexual female HB (*H.* sp. Midgley’s; ID: HB_3); (*D*) hybrid female *H.* sp. Midgley’s × *H.* sp. Bald (ID: HB×HX_5). According to the morphology of gonads, several cell types can be determined: S, spermatids; P, cells in the pachytene stage of meiotic division; G, germ cells; D, diplotene cells of meiotic division. Bars equal 50 µm.

Additionally, to investigate whether gametogenesis operates normally in hybrid males and females, we performed the analysis of gonadal microanatomy using confocal scanning microscopy in seven adult individuals. We checked one hybrid male and two hybrid females as well as three sexual males and one sexual females. The gonadal morphology of adult hybrids of both sexes is similar to those of sexual individuals (fig. 3). In hybrid males, we detected large clusters of spermatids. Smaller clusters were represented by cells during the pachytene stage of meiosis. Individual gonocytes, as well as primordial germ cells (PGC), were identified by immunostaining of Vasa protein. Gonads of adult females clearly showed diplotene oocytes as well as individual gonocytes located on the periphery of the gonad (fig. 3). In combination with results from DNA flow cytometry, we found no obvious differences between sexual and hybrid individuals, suggesting that the fertility of hybrid males and females is not reduced when compared with their sexual relatives.

### Genome Elimination Occurs in Juvenile Individuals

**Fig. 4.**
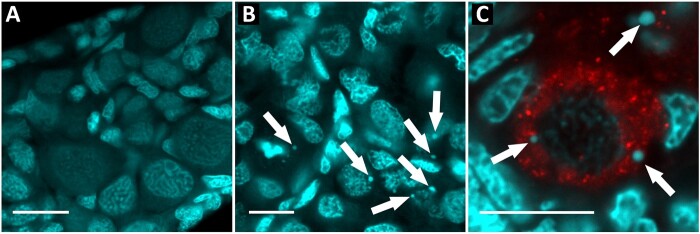
Comparison of gonadal microanatomy in sexual and hybrid juvenile individuals. Whole-mount immunofluorescent staining with antibodies against Vasa protein (red) identifying germ cells. DAPI is visualizing chromatin (cyan). (*A*) Sexual individual *H.* sp. Midgley’s (HB_6); (*B*) and (*C*) hybrid individuals *H.* sp. Midgley’s × *H.* sp. Bald (HB×HX_8 and HB×HX_9); arrows indicate micronuclei in the cytoplasm of germ cells. Bars equal 50 µm.

According to the analysis of chromosomal spreads from gonads of adult hybrid individuals and the absence of one parental set during meiosis, we looked for evidence whether genome elimination of one set followed by genome duplication of another set takes place prior to meiosis. In order to detect the process of genome elimination, we analyzed juvenile fish (before fully developed gonads, i.e., 1–2 months old). In gonads of two sexual and four juvenile hybrids, gonial cells were identified with antibodies against the Vasa protein as large cells with multiple nucleoli and less intensive chromatin staining compared with somatic cells (fig. 4). At this developmental stage, meiotic cells were isolated or absent, and most cells were gonial and actively dividing as we observed multiple mitotic divisions. In all observed hybrid individuals, we detected micronuclei in the cytoplasm of germ cells (fig. 4*B*). Micronuclei were presented as a round chromatin positive body, usually with the more intense chromatin staining, suggesting possible heterochromatinization (fig. 4*B* and C). The number of micronuclei varied from one to seven per individual germ cell with an average of four micronuclei per cell. In sexual species, we have not detected any micronuclei in fish of the same age. Our results suggest that genome elimination has already occurred via micronuclei formation before meiosis commences in juvenile carp gudgeons.

## Discussion

### The First Hybridogenetic Animal from the Southern Hemisphere

**Fig. 5.**
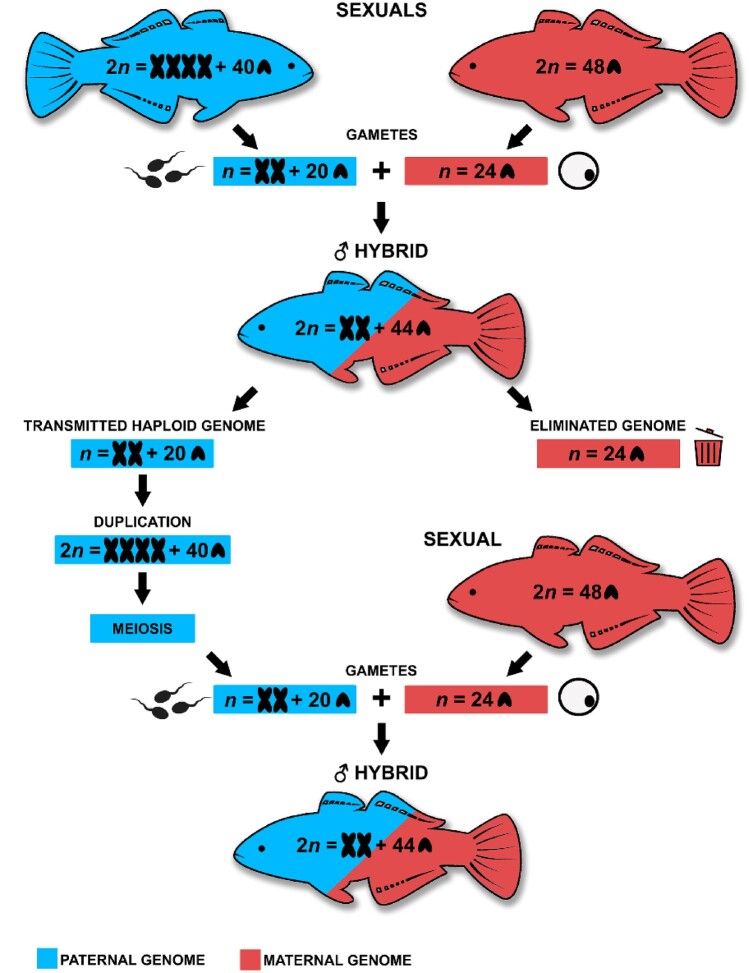
Schematic diagram of genome elimination in carp gudgeons. Diagram represents one of the case examples of this study.

Both sexual reproduction and uniparental genome elimination require fertilization, meiosis and formation of haploid gametes. However, only the uniparental genome elimination leads to segregation of the genomes nonrandomly, creating asymmetric genetic systems with uneven sex ratio as an evolutionary playground for peculiar phenotypes (Normark 2001; Austin et al. 2009; Ross et al. 2011). Our study has confirmed and delivered direct evidence for classic hybridogenesis as a reproductive mode for the unisexual Australian carp gudgeon hybrids (fig. 5), correctly predicted by Bertozzi et al. (2000) and later Schmidt et al. (2011). Apart from the well-known cases of unisexual reproduction, including hybridogenesis in the Northern Hemisphere, all the obligate unisexual animals from South America, Australia, and New Zealand appear to reproduce through parthenogenesis (i.e., “virgin birth”; reproduction without mating; Schön et al. 2009). Australian carp gudgeons add to the knowledge of the formation and global distribution of unisexual reproduction as the first-known animals using hybridogenesis in the Southern Hemisphere.

### Uniformity in Fertility and Ploidy Level but High Variability in Karyotypes

Most unisexual animal taxa reproduce as all-female populations, with males being typically absent or sterile. Undeveloped gonads and inability to produce sperm was frequently found among hybrids from various genera, for example, *Cobitis* loaches (Juchno et al. 2017; Juchno and Boroń 2018; Jablonska et al. 2020), *Misgurnus* loaches (Itono et al. 2006), oribatid mites (Heethoff et al. 2009), *Bacillus* stick insects (Mantovani and Scali 1992; Mantovani et al. 1999), or *Diadromus* wasps (El Agoze et al. 1994). In all these examples, hybrid females were able to reproduce normally and did not exhibit any gonadal aberrations. Previous studies have shown that the hybrid male’s sterility is caused by the inability to modify their gametogenesis in order to achieve clonality as females do. In this respect, males have problems with orthologous pairing in meiosis (Kuroda et al. 2019; Dedukh, Majtánová, et al. 2020; Spangenberg et al. 2017). Our analysis of gonadal microanatomy and flow cytometry did not indicate any aberration in male and female fertility, as both of them exhibited normal gonads with cells on various gametogenic stages.

In carp gudgeons, all observed hybrid males demonstrated the usual pairing of chromosomes during meiotic division (fig. 1). Thus, hybridogenetic reproduction does not restrain any gametogenic stages in males, and those can produce visually functional gametes (fig. 3). One comparative diploid system exists in Central Europe in water frogs, where the sexual species lives with the all-male hybrid lineages (Doležálková et al. 2016; Doležálková-Kaštánková et al. 2018). In these populations, male gametes are also produced hybridogenetically. Less surprisingly, ovarian microanatomy of carp gudgeons confirmed the functional gametogenesis for female hybrids, which produces oocytes (fig. 3). However, carp gudgeons represent an enigmatic model group, since the all-diploid ploidy level is linked with co-occurring hybrids of both sexes. Our flow cytometric and karyotype analyses showed no evidence of triploid individuals, which is in agreement with previous carp gudgeon studies (Bertozzi et al. 2000; Schmidt et al. 2011; Unmack et al. 2019). Indeed, sexual/unisexual complexes comprising strictly diploid hybrids are rare and were recently described in *Hexagrammos* fish populations (Suzuki et al. 2017). All other animal systems, in which both males and females can reproduce through mechanisms alternative to sexual reproduction, need the presence of polyploid individuals to be functional and stable (Alves et al. 2001; Scali et al. 2003; Stöck et al. 2012; Collares-Pereira et al. 2013; Dedukh et al. 2015; Zhang et al. 2015).

This study represents the first report of karyotype composition in sexual species and hybrid individuals in the fish genus *Hypseleotris*. We described intraspecific karyotype variability in sexual species and hybrids bearing their genomes. Surprisingly, our comparative analysis revealed a high level of karyotype variability within and between sexual species as well as hybrids (table 1). Karyotypes of these fishes included mostly a number of acrocentric chromosomes accompanied by metacentric chromosomes varying from zero to three across individuals. Variation in chromosome numbers has been frequently observed in hybrid fish taxa. However, the pattern was caused by leakage of individual chromosomes from sperm during gynogenesis, or due to aberrant cell divisions (Sola et al. 1992; Fontana et al. 2007; Zhang et al. 2015; Suzuki et al. 2017). Hybridization events may thus be one of the driving forces causing karyotype reorganization.

Despite the detected karyotype variability, the number of chromosome arms remains constant for all observed individuals, NF = 48. That suggests that presented metacentric chromosomes could arise via Robertsonian rearrangements involving the centric fusion of acrocentric chromosomes. Robertsonian translocations represent a relatively frequent phenomenon causing variability of chromosomal numbers within individuals of the same species. They were described in various fish species (Galetti et al. 2000; Morescalchi et al. 2011; Guyomard et al. 2012), and are considered to have generally little impact on meiotic chromosome pairing (Lanzone et al. 2007). Therefore, they might not represent a barrier to hybridization among individuals with different karyotypes (Lajus 2007).

### Evidence for Intact Parental Chromosomal Sets and Uniparental Inheritance in Hybrids

Using GISH, we identified two clear groups of chromosomal constituents in hybrid soma. The origin of haploid sets to the parental species from which they derive was difficult to trace back only at some individuals due to a limitation of GISH markers and intraspecific chromosomal variation within sexuals (fig. 2, table 1). Nevertheless, chromosomes were divergent enough in DNA sequence variation to bind labeled DNA species-specifically and split them into haploid sets, and the method is still powerful enough to detect possible intergenomic exchanges between chromosomes as found in unisexual salamanders (Bi et al. 2007). Studied carp gudgeon hybrids had a rather integral structure of parental sets, typical for well-maintained F_1_ hybrid constitutions like other gynogenetic fishes or hybridogenetic water frogs (Zaleśna et al. 2011; Majtánová et al. 2016). Similarly, the integral character of a single parental (species-specific) set in germ cells of several hybrid individuals (fig. 2) suggests that parental sets might have been formed clonally and their reproduction was hemiclonal.

Carp gudgeons are a remarkable group for its nonrandom pattern in uniparental chromosomal inheritance. All of those hybrids having one to three metacentrics in the soma also had the metacentric chromosomes in gonadal tissue, and in twice the number. We provide evidence that genome duplication occurs premeiotically and results in the uniparental hybridogenetic reproduction as previously suggested based on allozyme and microsatellites data (Bertozzi et al. 2000; Schmidt et al. 2011). Moreover, based on our results, it seems there is a correlation between the presence of metacentric chromosome(s) and the genome being transmitted (fig. 5). Uzzell et al. (1980) proposed a hypothesis for water frogs that one parental genome may contain factors responsible for the induction of hybridogenetic gametogenesis and its preferential transmission. Despite the fact the exact mechanism of genome elimination in carp gudgeons remains unknown, and such information can only be obtained from future long-term breeding experiments, the observed preferential propagation of a genome bearing the metacentric chromosomes is documented for the first time and requires further study to shed light on the mechanisms of selective genome elimination. As such, the evolution of metacentric chromosomes from the ancestral acrocentric carp gudgeons karyotype (2*n* = 48) and the actual mosaic variation and distribution pattern in the genus remain enigmatic and deserves more detailed study.

### Mechanism of Uniparental Genome Elimination in Carp Gudgeons

Our gonocyte analysis in adults did not detect any traces of DNA degradation or chromosomal lagging that usually accompany uniparental genome elimination in various organisms (Ishii et al. 2016; Chmielewska et al. 2018). It allowed us to infer that genome elimination does not occur in adult individuals. However, we detected micronuclei in the cytoplasm of germ cells in juvenile hybrids during stages of differentiation in which fish gonads contain somatic and germ cells. Germ cells that arise from PGC of an embryo actively proliferate, giving rise to primary oogonia and prespermatogonia (Van Winkoop et al. 1994). These ontogenetic stages usually correspond with the sexual differentiation of fish gonads. Micronuclei were shown to be connected with genome elimination in *Pelophylax* water frogs (Ogielska 1994; Chmielewska et al. 2018; Dedukh, Riumin, et al. 2020). These tetrapods gradually eliminate chromosomes through the accumulation of heterochromatin markers and degradation inside autophagosomes (Chmielewska et al. 2018; Dedukh, Riumin, et al. 2020). Such observations contrast with hybridogenetic *Poeciliopsis* females, which have variation in uniparental elimination modes. In this fish the whole paternal genome is eliminated during single oogonial division when attached to the unipolar spindle (Cimino 1972).

In carp gudgeons, we observed from one to seven micronuclei per cell, which is mechanistically closer to the gradual process of elimination rather than elimination all at once. The gradual genome elimination is a widespread pathway in eukaryotes, operating in plant hybrids, during programmed genome rearrangements in sea lampreys, or elimination of B chromosomes in insects and birds (Subrahmanyam and Kasha 1973; Gernand et al. 2006; Staiber 2006; Timoshevskiy et al. 2016; Torgasheva et al. 2019). The micronuclei are well-known structures that appear as a result of chromosome missegregation and reflect chromosome instability in many kinds of cancer cells (He et al. 2019), or a variety of cells exposed to genotoxic agents (Sánchez et al. 2000). Thus, the presence of micronuclei observed in hybrid individuals from carp gudgeons may indicate the gradual elimination of one of the parental genomes during their hybridogenetic reproduction.

In this article, we have presented cytological mechanisms underlying uniparental genome elimination and hybridogenesis in the Australian carp gudgeons. We anticipate that carp gudgeons will provide a good model system to help unveil some fundamental biological phenomena. A comparison of karyotypes provides the first view of the preferential transmission of the genome bearing the metacentric chromosomes. Chromosomal remodeling resulting in diverse karyotype variation seems to be linked with local hybridization and asexual reproduction when compared with a conservative karyotype of pure sexual populations free of hybrids. Second, there are not many groups of animal hybrids in which both sexes have functional gonads maintaining their reproductive potential. Carp gudgeons may, therefore, provide insights into conditions both for hybrid fertility and sterility. Finally, the occurrence of hybridogenesis in the Southern Hemisphere suggests that not only the geographic parthenogenesis (sensu Kearney 2005) is distributed worldwide, and may tell us more about the geography and demography in the unisexual origins.

## Supplementary Material

Supplementary data are available at *Genome Biology and Evolution* online.

## Supplementary Material

evab030_Supplementary_Data

## Notes

**Data deposition:** All data supporting our results and conclusions, along with sufficient details, are included in the manuscript and supplementary material, Supplementary Material online.

## References

[evab030-B1] Alves MJ , Coelho MM, Collares-Pereira MJ. 2001. Evolution in action through hybridisation and polyploidy in an Iberian freshwater fish: a genetic review. Genetica 111(1/3):375–385. 11841181 10.1023/a:1013783029921

[evab030-B2] Austin B , Trivers R, Burt A. 2009. Genes in conflict: the biology of selfish genetic elements. Cambridge (MA): Harvard University Press.

[evab030-B3] Bernstein H , Bernstein C. 2010. Evolutionary origin of recombination during meiosis. BioScience 60(7):498–505.

[evab030-B4] Bertollo LAC , Cioffi MdB, Moreira-Filho O. 2015. Fish cytogenetic techniques. In: Ozouf-Costaz C, Pisano E, Foresti F, Foresti de Almeida-Toledo L, editors. Direct chromosome preparation from freshwater teleost fishes. Boca Raton (FL): CRC Press. p. 21–26.

[evab030-B5] Bertozzi T , Adams M, Walker KF. 2000. Species boundaries in carp gudgeons (Eleotrididae: *Hypseleotris*) from the River Murray, South Australia: evidence for multiple species and extensive hybridization. Mar Freshwater Res. 51(8):805–815.

[evab030-B6] Bi K , Bogart JP, Fu J. 2007. Intergenomic translocations in unisexual salamanders of the genus *Ambystoma* (Amphibia, Caudata). Cytogenet Genome Res. 116(4):289–297. 17431327 10.1159/000100413

[evab030-B7] Carmona JA , Sanjur OI, Doadrio I, Machordom A, Vrijenhoek RC. 1997. Hybridogenetic reproduction and maternal ancestry of polyploid Iberian fish: the *Tropidophoxinellus alburnoides* complex. Genetics 146(3):983–993. 9215902 10.1093/genetics/146.3.983 PMC1208066

[evab030-B8] Chmielewska M , et al 2018. The programmed DNA elimination and formation of micronuclei in germ line cells of the natural hybridogenetic water frog *Pelophylax esculentus*. Sci Rep. 8:1–19. 29777142 10.1038/s41598-018-26168-z PMC5959867

[evab030-B9] Cimino MC. 1972. Egg-production, polyploidization and evolution in a diploid all-female fish of the genus *Poeciliopsis*. Evolution 26(2):294–306. 28555744 10.1111/j.1558-5646.1972.tb00195.x

[evab030-B10] Collares-Pereira MJ , Matos I, Morgado-Santos M, Coelho MM. 2013. Natural pathways towards polyploidy in animals: the *Squalius alburnoides* fish complex as a model system to study genome size and genome reorganization in polyploids. Cytogenet Genome Res. 140(2–4):97–116. 23796598 10.1159/000351729

[evab030-B11] Comai L. 2014. Genome elimination: translating basic research into a future tool for plant breeding. PLoS Biol. 12(6):e1001876. 24915001 10.1371/journal.pbio.1001876 PMC4051579

[evab030-B12] Crow JF , Kimura M. 1965. Evolution in sexual and asexual populations. Am Nat. 99(909):439–450.

[evab030-B13] Dedukh D , et al 2015. Optional endoreplication and selective elimination of parental genomes during oogenesis in diploid and triploid hybrid European water frogs. PLoS One 10(4):e0123304. 25894314 10.1371/journal.pone.0123304 PMC4403867

[evab030-B14] Dedukh D , Litvinchuk S, Rosanov J, Shabanov D, Krasikova A. 2017. Mutual maintenance of di-and triploid *Pelophylax esculentus* hybrids in RE systems: results from artificial crossings experiments. BMC Evol Biol. 17:1–15. 29041900 10.1186/s12862-017-1063-3 PMC5645918

[evab030-B15] Dedukh D , Majtánová Z, et al 2020. Parthenogenesis as a solution to hybrid sterility: the mechanistic basis of meiotic distortions in clonal and sterile hybrids. Genetics 215(4):975–987. 32518062 10.1534/genetics.119.302988 PMC7404241

[evab030-B16] Dedukh D , Riumin S, et al 2020. Micronuclei in germ cells of hybrid frogs from *Pelophylax esculentus* complex contain gradually eliminated chromosomes. Sci Rep. 10(1):8720. 32457346 10.1038/s41598-020-64977-3 PMC7251083

[evab030-B17] Doležálková M , et al 2016. Is premeiotic genome elimination an exclusive mechanism for hemiclonal reproduction in hybrid males of the genus *Pelophylax*? BMC Genet. 17(1):100. 27368375 10.1186/s12863-016-0408-z PMC4930623

[evab030-B18] Doležálková-Kaštánková M , et al 2018. All-male hybrids of a tetrapod *Pelophylax esculentus* share its origin and genetics of maintenance. Biol Sex Diff. 9:13. 10.1186/s13293-018-0172-z PMC5880063 29609661

[evab030-B19] El Agoze M , Drezen JM, Renault S, Periquet G. 1994. Analysis of the reproductive potential of diploid males in the wasp *Diadromus pulchellus* (Hymenoptera: Ichneumonidae). Bull Entomol Res. 84(2):213–218.

[evab030-B20] Fontana F , Zane L, Congiu PAL. 2007. Polyploidy in Acipenseriformes: cytogenetic and molecular approaches. In: Pisano E, Ozouf-Costaz C, Foresti F, Kapoor BG, editors. Fish Cytogenetics. Enfield (England): Science Publisher. p. 385–403.

[evab030-B21] Galetti PM , Aguilar CT, Molina WF. 2000. An overview of marine fish cytogenetics. Hydrobiologia 420(1):55–62.

[evab030-B22] Gardner A , Ross L. 2014. Mating ecology explains patterns of genome elimination. Ecol Lett. 17(12):1602–1612. 25328085 10.1111/ele.12383 PMC4240462

[evab030-B23] Gernand D , Rutten T, Pickering R, Houben A. 2006. Elimination of chromosomes in *Hordeum vulgare* × *H. bulbosum* crosses at mitosis and interphase involves micronucleus formation and progressive heterochromatinization. Cytogenet Genome Res. 114(2):169–174. 16825770 10.1159/000093334

[evab030-B24] Gruber B , Unmack PJ, Berry OF, Georges A. 2018. DARTR: an R package to facilitate analysis of SNP data generated from reduced representation genome sequencing. Mol Ecol Resour. 18(3):691–699. 29266847 10.1111/1755-0998.12745

[evab030-B25] Guyomard R , Boussaha M, Krieg F, Hervet C, Quillet E. 2012. A synthetic rainbow trout linkage map provides new insights into the salmonid whole genome duplication and the conservation of synteny among teleosts. BMC Genet. 13:15. 22424132 10.1186/1471-2156-13-15 PMC3368724

[evab030-B26] He B , et al 2019. Chromosomes missegregated into micronuclei contribute to chromosomal instability by missegregating at the next division. Oncotarget 10(28):2660–2674. 31105868 10.18632/oncotarget.26853 PMC6505630

[evab030-B27] Heethoff M , Norton RA, Scheu S, Maraun M. 2009. Parthenogenesis in oribatid mites (Acari, Oribatida): evolution without sex. In: Lost sex. New York: Springer. p. 241–257.

[evab030-B28] Heppich S , Tunner HG, Greilhuber J. 1982. Premeiotic chromosome doubling after genome elimination during spermatogenesis of the species hybrid *Rana esculenta*. Theor Appl Genet. 61(2):101–104. 24270328 10.1007/BF00273874

[evab030-B29] Hoese DF , Larson HK, Llewellyn LC. 1980. Family Eleotridae: gudgeons. In: Freshwater fishes of Southeastern Australia. Sydney (Australia): Reed Pty Ltd. p. 167–185.

[evab030-B30] Ishii T , Karimi-Ashtiyani R, Houben A. 2016. Haploidization via chromosome elimination: means and mechanisms. Annu Rev Plant Biol. 67:421–438. 26772657 10.1146/annurev-arplant-043014-114714

[evab030-B31] Itono M , et al 2006. Premeiotic endomitosis produces diploid eggs in the natural clone loach, *Misgurnus anguillicaudatus* (Teleostei: Cobitidae). J Exp Zool A Comp Exp Biol. 305(6):513–523. 16526047 10.1002/jez.a.283

[evab030-B32] Jablonska O , Juchno D, Leska A, Kowalewska K, Boroń A. 2020. Variable occurrence of apoptosis in the testes of diploid and sterile allotetraploid *Cobitis* (Teleostei, Cobitidae) males during the reproductive cycle. J Exp Biol. 223(9):jeb212050. 32205361 10.1242/jeb.212050

[evab030-B33] Juchno D , Boroń A. 2018. Histological evidence that diploid hybrids of *Cobitis taenia* and *C. elongatoides* (Teleostei, Cobitidae) develop into fertile females and sterile males. Hydrobiologia 814(1):147–159.

[evab030-B34] Juchno D , et al 2017. Evidence of the sterility of allotetraploid *Cobitis* loaches (Teleostei, Cobitidae) using testes ultrastructure. J Exp Zool A Ecol Integr Physiol. 327(1):66–74. 29356377 10.1002/jez.2071

[evab030-B35] Kearney M. 2005. Hybridization, glaciation and geographical parthenogenesis. Trends Ecol Evol. 20(9):495–502. 16701426 10.1016/j.tree.2005.06.005

[evab030-B36] Kilian A , et al 2012. Diversity arrays technology: a generic genome profiling technology on open platforms. Methods Mol Biol. 888:67–89. 22665276 10.1007/978-1-61779-870-2_5

[evab030-B37] Kimura‐Kawaguchi MR , et al 2014. Identification of hemiclonal reproduction in three species of *Hexagrammos* marine reef fishes. J Fish Biol. 85:189–209. 24903212 10.1111/jfb.12414

[evab030-B38] Kondrashov AS. 1988. Deleterious mutations and the evolution of sexual reproduction. Nature 336(6198):435–440. 3057385 10.1038/336435a0

[evab030-B39] Kuroda M , Fujimoto T, Murakami M, Yamaha E, Arai K. 2019. Aberrant meiotic configurations cause sterility in clone-origin triploid and inter-group hybrid males of the Dojo Loach, *Misgurnus anguillicaudatus*. Cytogenet Genome Res. 158(1):46–54. 31158836 10.1159/000500303

[evab030-B40] Lajus D. 2007. Chromosomal analysis in population structuring and stock identification: Robertsonian polymorphism in the White Sea herring (*Clupea pallasi marisalbi*). In: Pisano E, editor. Fish cytogenetics. Boca Raton (FL): CRC Press. p. 261–287.

[evab030-B41] Lanzone C , Giménez MD, Santos JL, Bidau CJ. 2007. Meiotic effects of Robertsonian translocations in tuco-tucos of the *Ctenomys perrensi superspecies* (Rodentia: Ctenomyidae). Caryologia 60(3):233–244.

[evab030-B42] Lavanchy G , Schwander T. 2019. Hybridogenesis. Curr Biol. 29(1):R9–R11. 30620918 10.1016/j.cub.2018.11.046

[evab030-B43] Lenormand T , Engelstädter J, Johnston SE, Wijnker E, Haag CR. 2016. Evolutionary mysteries in meiosis. Philos Trans R Soc B. 371(1706):20160001. 10.1098/rstb.2016.0001 PMC5031626 27619705

[evab030-B44] Majtánová Z , et al 2016. Asexual reproduction does not apparently increase the rate of chromosomal evolution: karyotype stability in diploid and triploid clonal hybrid fish (*Cobitis*, Cypriniformes, Teleostei). PLoS ONE 11(1):e0146872. 26808475 10.1371/journal.pone.0146872 PMC4726494

[evab030-B45] Mantovani B , Passamonti M, Scali V. 1999. Genomic evolution in parental and hybrid taxa of the genus *Bacillus* (Insecta, Phasmatodea). Ital J Zool. 66(3):265–272.

[evab030-B46] Mantovani B , Scali V. 1992. Hybridogenesis and androgenesis in the stick‐insect *Bacillus rossius*‐*grandii benazzii* (Insecta, Phasmatodea). Evolution 46(3):783–796. 28568678 10.1111/j.1558-5646.1992.tb02084.x

[evab030-B47] Morescalchi MA , Stingo V, Capriglione T. 2011. Cytogenetic analysis in *Polypterus ornatipinnis* (Actinopterygii, Cladistia, Polypteridae) and 5S rDNA. Mar Genomics. 4(1):25–31. 21429462 10.1016/j.margen.2010.12.002

[evab030-B48] Munehara H , Horita M, Kimura‐Kawaguchi MR, Yamazaki A. 2016. Origins of two hemiclonal hybrids among three *Hexagrammos* species (Teleostei: Hexagrammidae): genetic diversification through host switching. Ecol Evol. 6(19):7126–7140. 28725387 10.1002/ece3.2446 PMC5513241

[evab030-B49] Nabais C , Pereira C, Cuñado N, Collares-Pereira MJ. 2012. Synaptonemal complexes in the hybridogenetic *Squalius alburnoides* fish complex: new insights on the gametogenesis of allopolyploids. Cytogenet Genome Res. 138(1):31–35. 22796718 10.1159/000339522

[evab030-B50] Normark BB. 2001. Genetic conflict and the dizygotic soma: on the adaptive significance of polar body transmission and the polyploid bacteriome in Pseudococcidae and Diaspididae. Boll Lab Entomol Agrar Portici. 57:151–160.

[evab030-B51] Ogielska M. 1994. Nucleus-like bodies in gonial cells of *Rana esculenta* [Amphibia, Anura] tadpoles—a putative way of chromosome elimination. Zool Pol. 39:3–4.

[evab030-B52] Petronczki M , Siomos MF, Nasmyth K. 2003. Un ménage à quatre: the molecular biology of chromosome segregation in meiosis. Cell 112(4):423–440. 12600308 10.1016/s0092-8674(03)00083-7

[evab030-B53] Ross L , Shuker DM, Pen I. 2011. The evolution and suppression of male suicide under paternal genome elimination. Evolution 65(2):554–563. 21029080 10.1111/j.1558-5646.2010.01148.x

[evab030-B54] Sánchez P , Llorente MT, Castaño A. 2000. Flow cytometric detection of micronuclei and cell cycle alterations in fish-derived cells after exposure to three model genotoxic agents: mitomycin C, vincristine sulfate and benzo (a) pyrene. Mutat Res Genet Toxicol Environ Mutagen. 465(1–2):113–122. 10.1016/s1383-5718(99)00218-1 10708976

[evab030-B55] Sanei M , Pickering R, Kumke K, Nasuda S, Houben A. 2011. Loss of centromeric histone H3 (CENH3) from centromeres precedes uniparental chromosome elimination in interspecific barley hybrids. Proc Natl Acad Sci USA. 108(33):E498–E505. 21746892 10.1073/pnas.1103190108 PMC3158150

[evab030-B56] Scali V , Passamonti M, Marescalchi O, Mantovani B. 2003. Linkage between sexual and asexual lineages: genome evolution in *Bacillus* stick insects. Biol J Linn Soc. 79(1):137–150.

[evab030-B57] Schmidt DJ , Bond NR, Adams M, Hughes JM. 2011. Cytonuclear evidence for hybridogenetic reproduction in natural populations of the Australian carp gudgeon (*Hypseleotris*: Eleotridae). Mol Ecol. 20(16):3367–3380. 21777320 10.1111/j.1365-294X.2011.05206.x

[evab030-B58] Schön I , Martens K, van Dijk P. 2009. Lost sex. The evolutionary biology of parthenogenesis. Netherlands: Springer.

[evab030-B59] Schultz RJ. 1961. Reproductive mechanism of unisexual and bisexual strains of the viviparous fish *Poeciliopsis*. Evolution 15(3):302–325.

[evab030-B60] Sola L , Rossi AR, Iaselli V, Rasch EM, Monaco PJ. 1992. Cytogenetics of bisexual/unisexual species of *Poecilia*. II. Analysis of heterochromatin and nucleolar organizer regions in *Poecilia mexicana mexicana* by C-banding and DAPI, quinacrine, chromomycin A3, and silver staining. Cytogenet Cell Genet. 60(3–4):229–235. 1380417 10.1159/000133346

[evab030-B61] Spangenberg V , et al 2017. Reticulate evolution of the rock lizards: meiotic chromosome dynamics and spermatogenesis in diploid and triploid males of the genus *Darevskia*. Genes 8(6):149. 10.3390/genes8060149 PMC5485513 28538689

[evab030-B62] Staiber W. 2006. Chromosome elimination in germ line–soma differentiation of *Acricotopus lucidus* (Diptera, Chironomidae). Genome 49(3):269–274. 16604110 10.1139/g05-103

[evab030-B63] Stenberg P , Saura A. 2009. Cytology of asexual animals. In: Schön I, Martens K, Dijk P, editors. Lost sex. Netherlands: Springer. p. 63–74.

[evab030-B64] Stöck M , et al 2012. Simultaneous Mendelian and clonal genome transmission in a sexually reproducing, all-triploid vertebrate. Proc Biol Sci. 279(1732):1293–1299. 21993502 10.1098/rspb.2011.1738 PMC3282369

[evab030-B65] Subrahmanyam NC , Kasha KJ. 1973. Selective chromosomal elimination during haploid formation in barley following interspecific hybridization. Chromosoma 42(2):111–125.

[evab030-B66] Suwa K , Yamashita M. 2007. Regulatory mechanisms of oocyte maturation and ovulation. In: The fish oocyte. New York: Springer. p. 323–347.

[evab030-B67] Suzuki S , Arai K, Munehara H. 2017. Karyological evidence of hybridogenesis in Greenlings (Teleostei: Hexagrammidae). PLoS ONE 12(7):e0180626. 28678883 10.1371/journal.pone.0180626 PMC5498075

[evab030-B68] Thacker C , Unmack PJ. 2005. Phylogeny and biogeography of the eleotrid genus *Hypseleotris* (Teleostei: Gobioidei: Eleotridae), with redescription of *H. cyprinoides*. Rec Aust Mus. 57(1):1–13.

[evab030-B69] Timoshevskiy VA , Herdy JR, Keinath MC, Smith JJ. 2016. Cellular and molecular features of developmentally programmed genome rearrangement in a vertebrate (sea lamprey: *Petromyzon marinus*). PLoS Genet. 12(6):e1006103. 27341395 10.1371/journal.pgen.1006103 PMC4920378

[evab030-B70] Torgasheva AA , et al 2019. Germline-restricted chromosome (GRC) is widespread among songbirds. Proc Natl Acad Sci USA. 116(24):11845–11850. 31036668 10.1073/pnas.1817373116 PMC6575587

[evab030-B71] Tunner HG. 2009. Die klonale Struktur einer Wasserfroschpopulation 1. J Zool Syst Evol Res. 12(1):309–314.

[evab030-B72] Tunner HG , Heppich S. 1981. Premeiotic genome exclusion during oogenesis in the common edible frog, *Rana esculenta*. Naturwissenschaften 68(4):207–208. 6974310 10.1007/BF01047207

[evab030-B73] Unmack PJ. 2000. The genus *Hypseleotris* of southeastern Australia: its identification and breeding biology. Fish Sahul. 14:645–657.

[evab030-B74] Unmack PJ , et al 2019. Perspectives on the clonal persistence of presumed ‘ghost’ genomes in unisexual or allopolyploid taxa arising via hybridization. Sci Rep. 9(1):4730. 30894575 10.1038/s41598-019-40865-3 PMC6426837

[evab030-B75] Uzzell T , Hotz H, Berger L. 1980. Genome exclusion in gametogenesis by an interspecific *Rana* hybrid: evidence from electrophoresis of individual oocytes. J Exp Zool. 214(3):251–259.

[evab030-B76] Van Winkoop A , Timmermans LPM, Goos HT. 1994. Stimulation of gonadal and germ cell development in larval and juvenile carp (*Cyprinus carpio* L.) by homologous pituitary extract. Fish Physiol Biochem. 13(2):161–171. 24202315 10.1007/BF00004341

[evab030-B77] Zaleśna A , et al 2011. Evidence for integrity of parental genomes in the diploid hybridogenetic water frog *Pelophylax esculentus* by genomic *in situ* hybridization. Cytogenet Genome Res. 134(3):206–212. 21555873 10.1159/000327716

[evab030-B78] Zhang J , et al 2015. Meiosis completion and various sperm responses lead to unisexual and sexual reproduction modes in one clone of polyploid *Carassius gibelio*. Sci Rep. 5:10898. 26042995 10.1038/srep10898 PMC4455247

[evab030-B79] Zhang Q , Arai K, Yamashita M. 1998. Cytogenetic mechanisms for triploid and haploid egg formation in the triploid loach *Misgurnus anguillicaudatus*. J Exp Zool. 281(6):608–619.

