# Supplementary material for: Uniparental Genome Elimination in Australian Carp Gudgeons
Source: Genome Biol Evol. 2021 Feb 16;13(6):evab030. doi: 10.1093/gbe/evab030 (PMC8245195; doi:10.1093/gbe/evab030)
Supplement: evab030_Supplementary_Data [file evab030_supplementary_data.zip › Supplementary data_Rev2_clean copy.docx]

**SUPPLEMENTARY MATERIALS**

**Supplementary Methods**

**Taxonomic sampling.** As part of our research into this group of fishes we have established a reference set of 82 individuals spanning the taxonomic and geographic diversity of the hemiclonal complex plus *Hypseleotris klunzingeri*. Each individual has been sequenced for SNPs, allozymes and mtDNA in order to provide robust identifications. To this reference set we added 23 additional individuals successfully genotyped from this study to establish their identifications.

**SNP genotyping and data filtering.** Sequencing for the SNP dataset was performed using DArTseq™ (DArT Pty Ltd, Canberra Australia) using low density coverage, a variation of the double-digest RAD technique which combines next generation sequencing, complexity reduction using restriction enzymes, and implicit fragment size selection, as described by Kilian et al. (2012). Complexity reduction used the restriction enzymes SbfI (recognition sequence 5′-CCTGCA|GG-3′) and SphI (5’-GCATG|C-3’). All details of the sequencing methods used follow Georges et al. (2018).

The SNP dataset underwent two phases of filtering and error-checking, one included automatically as part of DArTseq™ standard protocols (fully detailed by Georges et al. 2018) followed by various operator-defined choices implemented on the final ‘raw dataset’ using the R-package dartR 2.1.3 (Gruber et al. 2018). These raw data were subjected to three sequential filtering procedures to generate two final SNP datasets, one with all taxa included, and a second one with only the hemiclonal complex species included to ensure that the few *H. klunzingeri* individuals were not causing any issues. For both datasets, these filters removed: (a) loci that did not show 100% reproducibility (RepAvg = 1) for the ~30% of individuals that are routinely randomly re-sequenced by DArT, (b) loci displaying more than 50% missing genotypes (callrate loc > 0.5), and (c) monomorphic loci in the second dataset which had *H. klunzingeri* removed.

**Identifications of individuals.** We used two approaches to identify individuals from this study. The first used Principal Coordinates Analysis (PCoA), as implemented in dartR (gl.pcoa and gl.pcoa.plot functions), to visualize the genetic affinities among individuals with no priors. The second method used the individual SNPs concatenated and examined using a Neighbor-Joining tree in PAUP* 4.0b10 (Swofford 2002).

**Supplementary Results**

SNP filtering

The initial dataset consisted of 2,302 SNPs and 105 individual fish. For the first dataset which included *H. klunzingeri* filtering for RepAve = 1 reduced the number of loci by 588, with 1,714 loci retained, followed by removing loci with >50% missing data resulted in an additional drop of 943 loci, with 771 loci retained for the final analyses. The second dataset which excluded *H. klunzingeri* started with 1,929 loci and 86 individuals. Filtering for RepAve = 1 reduced the number of loci by 547, with 1,382 loci retained, followed by removing loci with >50% missing data resulted in a drop of 651 loci, with 731 loci retained for the final analyses.

PCoA

Both PCoA plots provided clear separation between all of the taxa (seven and six, respectively) with over 45% of the variation explained in the first two axes. Both Neighbor-Joining trees (Supplementary Fig. S2 and S3) successfully placed all individuals into groups consistent with the PCoA results (Supplementary Fig. S4 and S5).

**Supplementary References**

Georges A, et al. 2018. Genomewide SNP markers breathe new life into phylogeography and species delimitation for the problematic short‐necked turtles (Chelidae: Emydura) of eastern Australia. Mol Ecol. 27:5195–5213.

Gruber B, Unmack PJ, Berry OF, Georges A. 2018. dartr: An r package to facilitate analysis of SNP data generated from reduced representation genome sequencing. Mol Ecol Res. 18:691–699.

Kilian A, et al. 2012. Diversity arrays technology: a generic genome profiling technology on open platforms. Methods Mol Biol. 888:67–89.

Swofford DL. 2002. *PAUP: phylogenetic analysis using parsimony, version 4.0 b10*. Sinauer Associates, Sunderland, MA.

**Supplementary Table S1.** Individuals used in this study

Notes: Codes refer to sexual species, *H. galii* (HA), *H*. sp. Midgley’s (HB), *H*. sp. Bald (HX) and *H. klunzingeri* (HK) and two interspecific F_1_hybrid genotypes designated as HA×HB and HB×HX.

**Supplementary Figures**

**
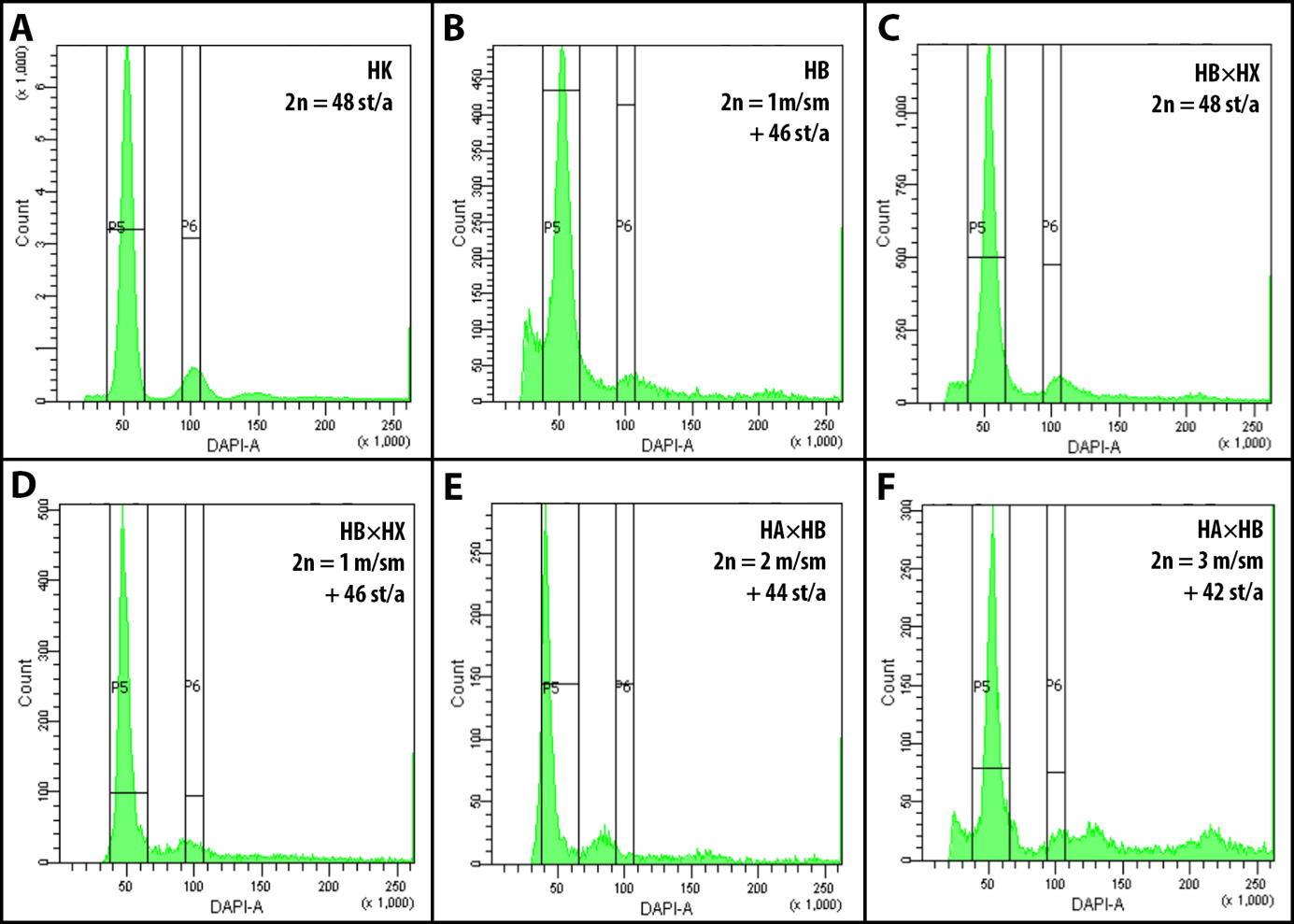
**

**Supplementary Fig. S1.** The measurement of ploidy levels of testes from sexual and hybrid individuals. (A, B) sexual males *H. klunzingeri* (HK) and *H*. sp. Midgley’s (HB); (C, D) hybrid genotypes HB×HX (*H*. sp. Midgley’s × *H*. sp. Bald); (E, F) hybrid genotypes HA×HB (*H. galii* × *H*. sp. Midgley’s). Chromosome composition of each individual is provided in the graphs (m/sm, meta-submetacentric; st/a, subtelocentric-acrocentric chromosomes). Both hybrid and sexual individuals possessed the most prominent 1C spermatids and spermatozoa, diploid 2C cells, and minor diploid 4C cells.


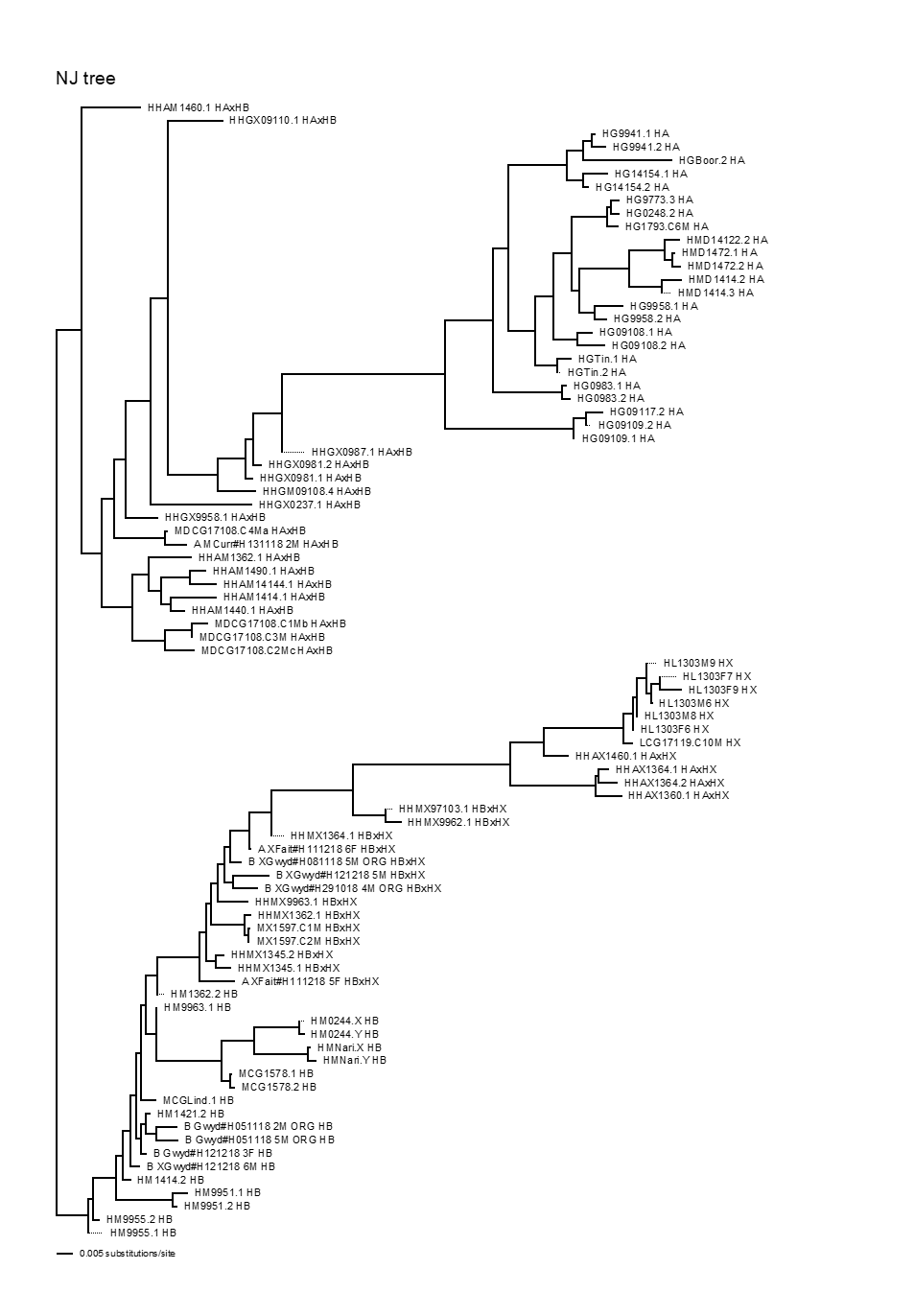


**Supplementary Fig. S2.** Neighbor-Joining tree based on SNPs dataset of 105 *Hypseleotris* individuals. Neighbor-Joining tree placed all individuals into groups consistent with the PCoA results.


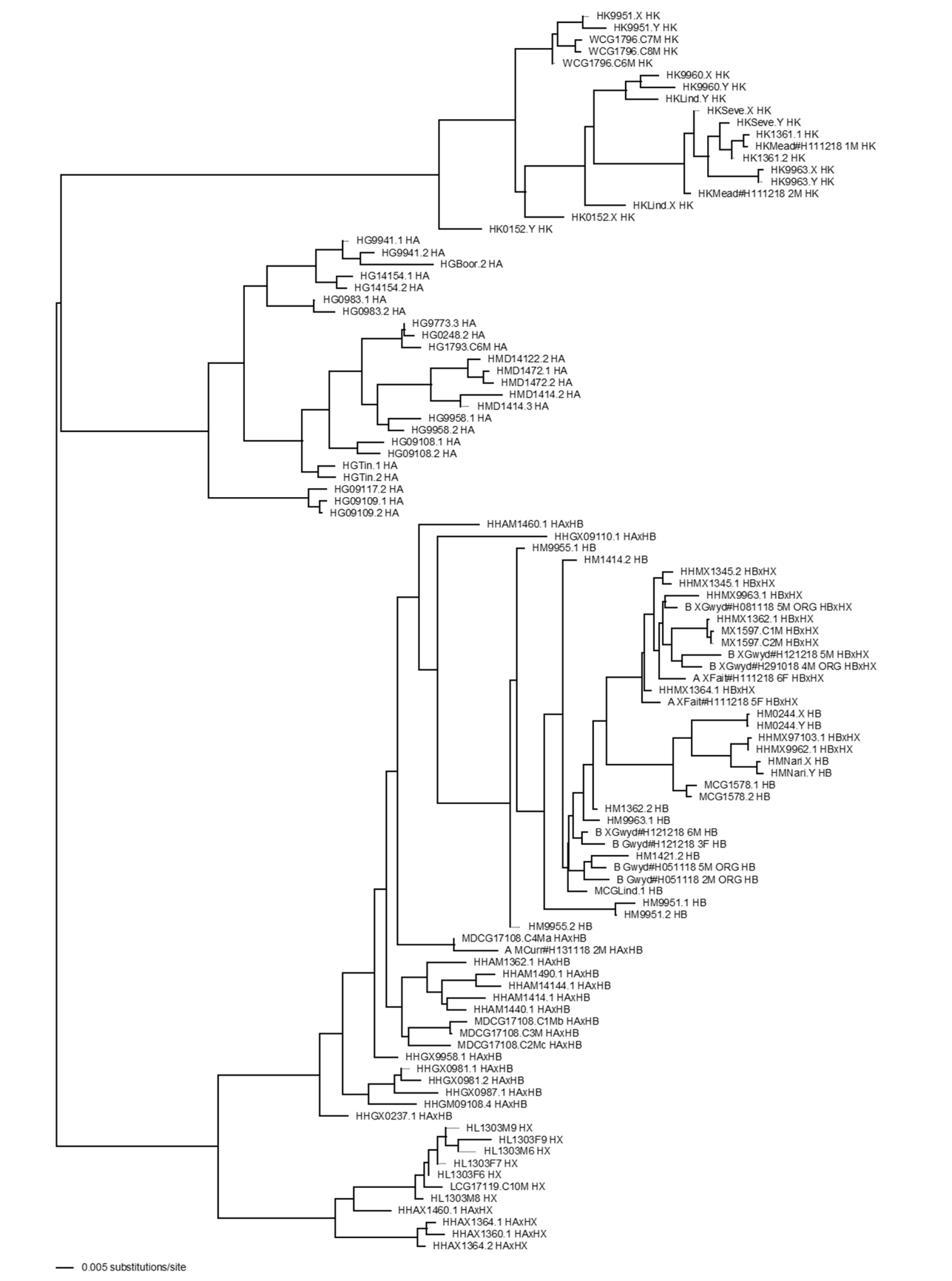


**Supplementary Fig. S3.** Neighbor-Joining tree based on SNPs dataset of 86 *Hypseleotris* individuals (*H. klunzingeri* excluded). Neighbor-Joining tree placed all individuals into groups consistent with the PCoA results.


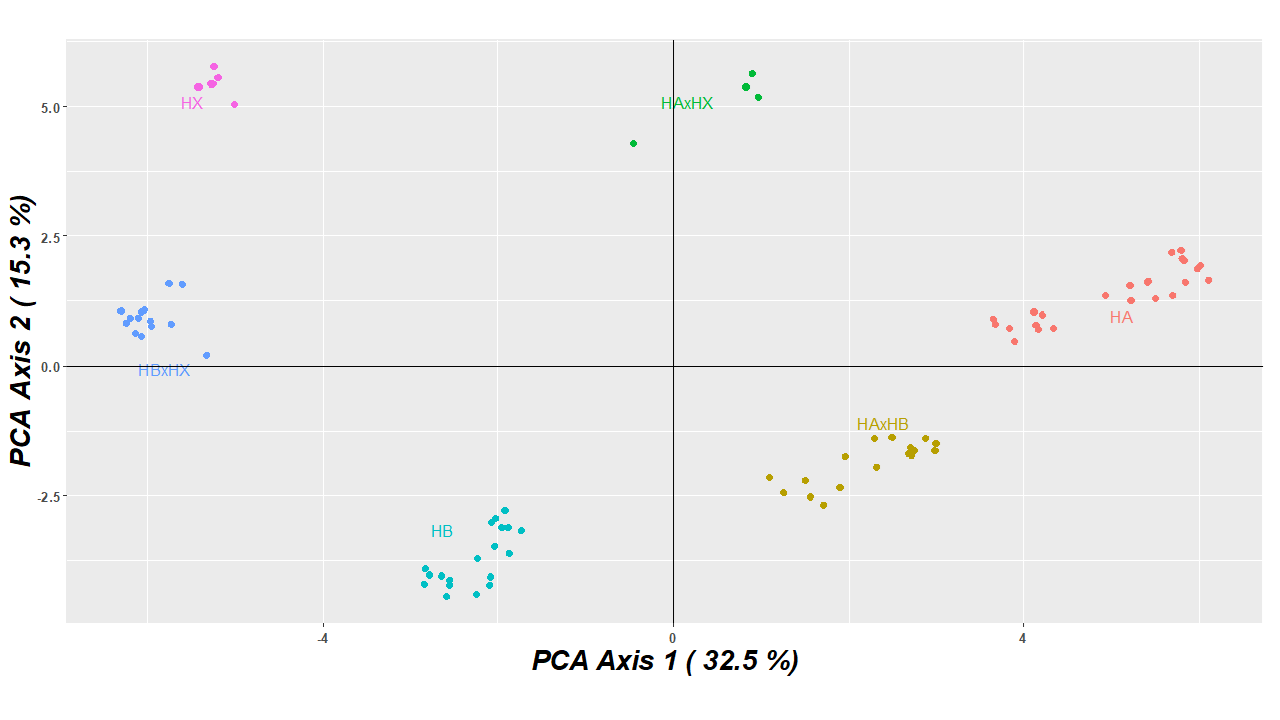


**Supplementary Fig. S4.** Scatterplot of the relative scores in the first two dimensions for the Principal Co-ordinates Analysis (PCoA) of 86 *Hypseleotris* individuals (*H. klunzingeri* excluded), based on the filtered SNP dataset. The scatterplot plot provided a clear separation between all of the six taxa. Three sexual species *H. galii* (HA), H. sp. Midgley’s (HB), and H. sp. Bald (HX), and three interspecific hybrid genotypes designated as HA×HB, HB×HX, and HA×HX (not analysed cytogenetically in this study).


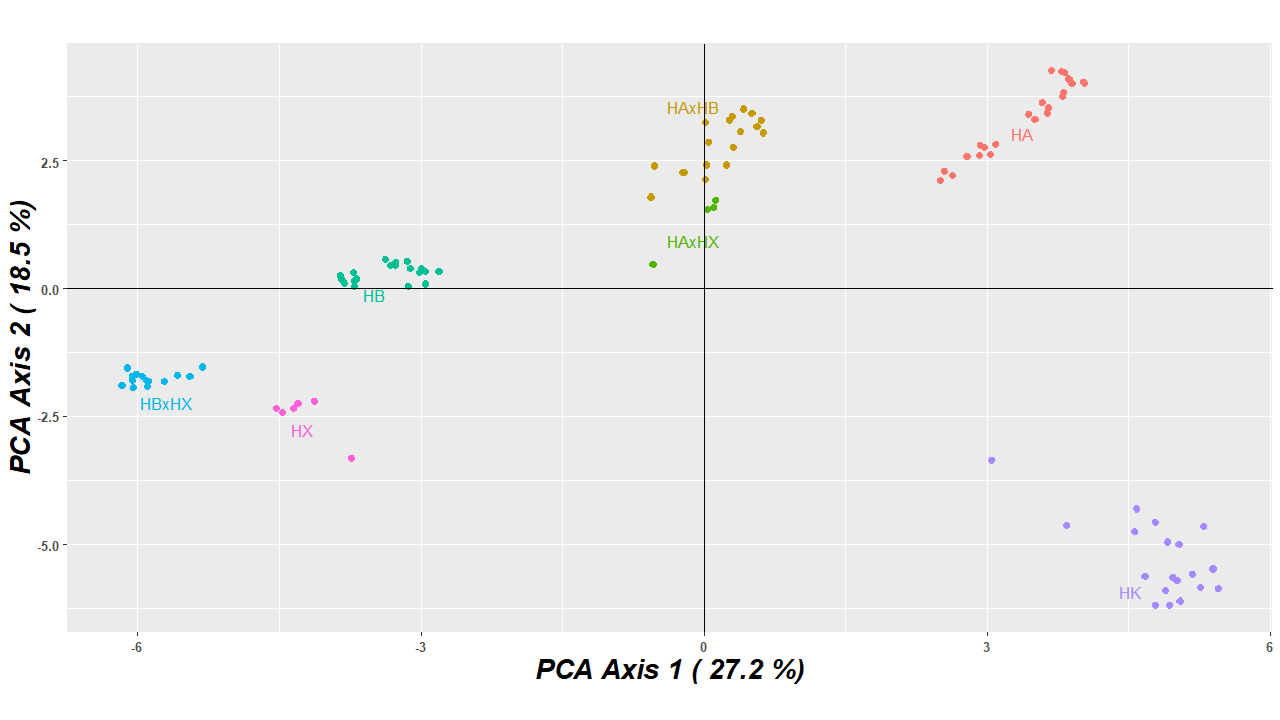


**Supplementary Fig. S5.** Scatterplot of the relative scores in the first two dimensions for the Principal Co-ordinates Analysis (PCoA) of 105 *Hypseleotris*, based on the filtered SNP dataset. The scatterplot plot provided a clear separation between all of the seven taxa. Four sexual species *H. galii* (HA), H. sp. Midgley’s (HB), H. sp. Bald (HX) and *H. klunzingeri* (HK) and three interspecific hybrid genotypes designated as HA×HB, HB×HX, and HA×HX (not analysed cytogenetically in this study).
