# Supplementary figures and images for: Uniparental Genome Elimination in Australian Carp Gudgeons
Source: Genome Biol Evol. 2021 Feb 16;13(6):evab030. doi: 10.1093/gbe/evab030 (PMC8245195; doi:10.1093/gbe/evab030)

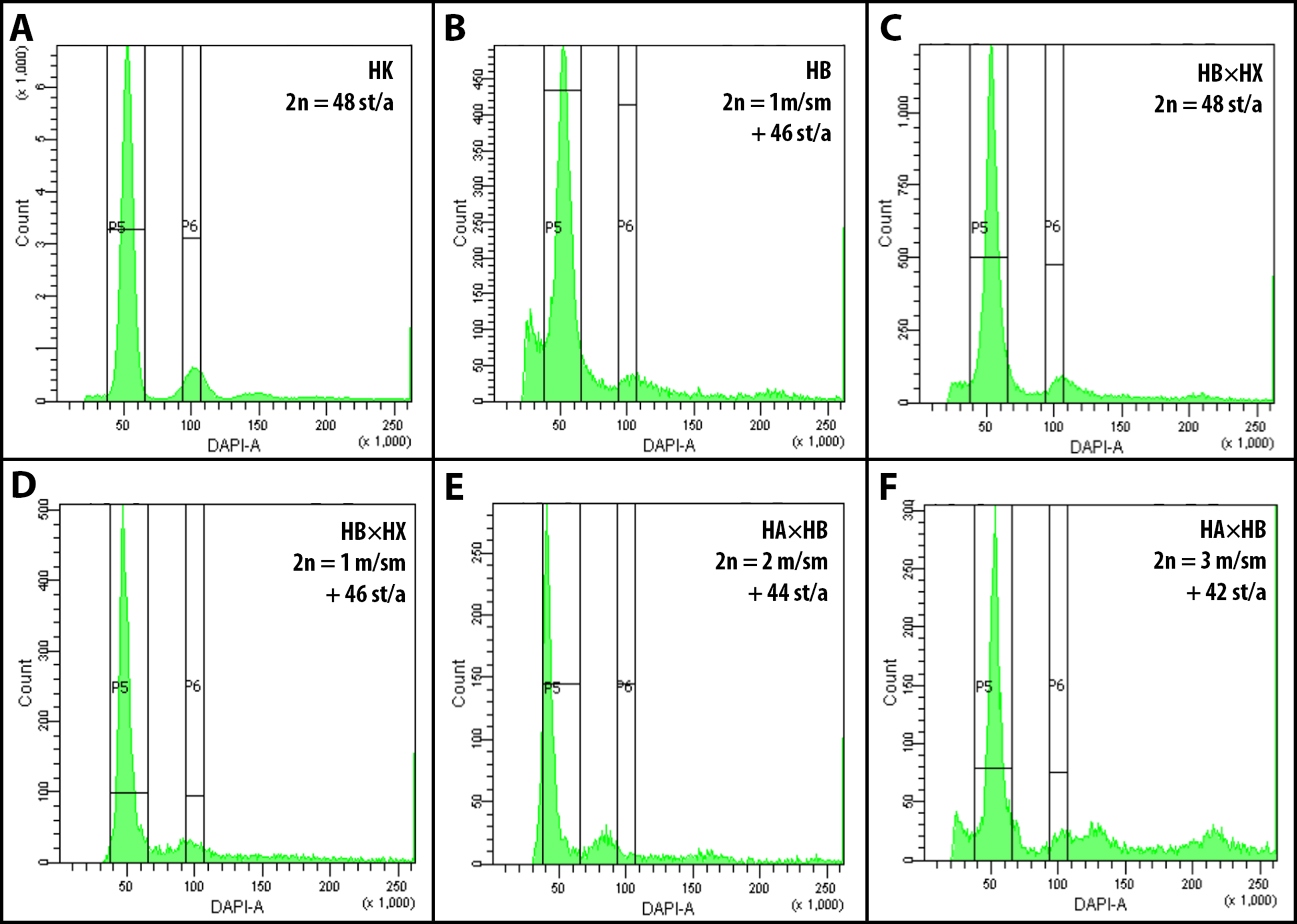

Supplement: evab030_Supplementary_Data [file evab030_supplementary_data.zip › Figure S1.tif]

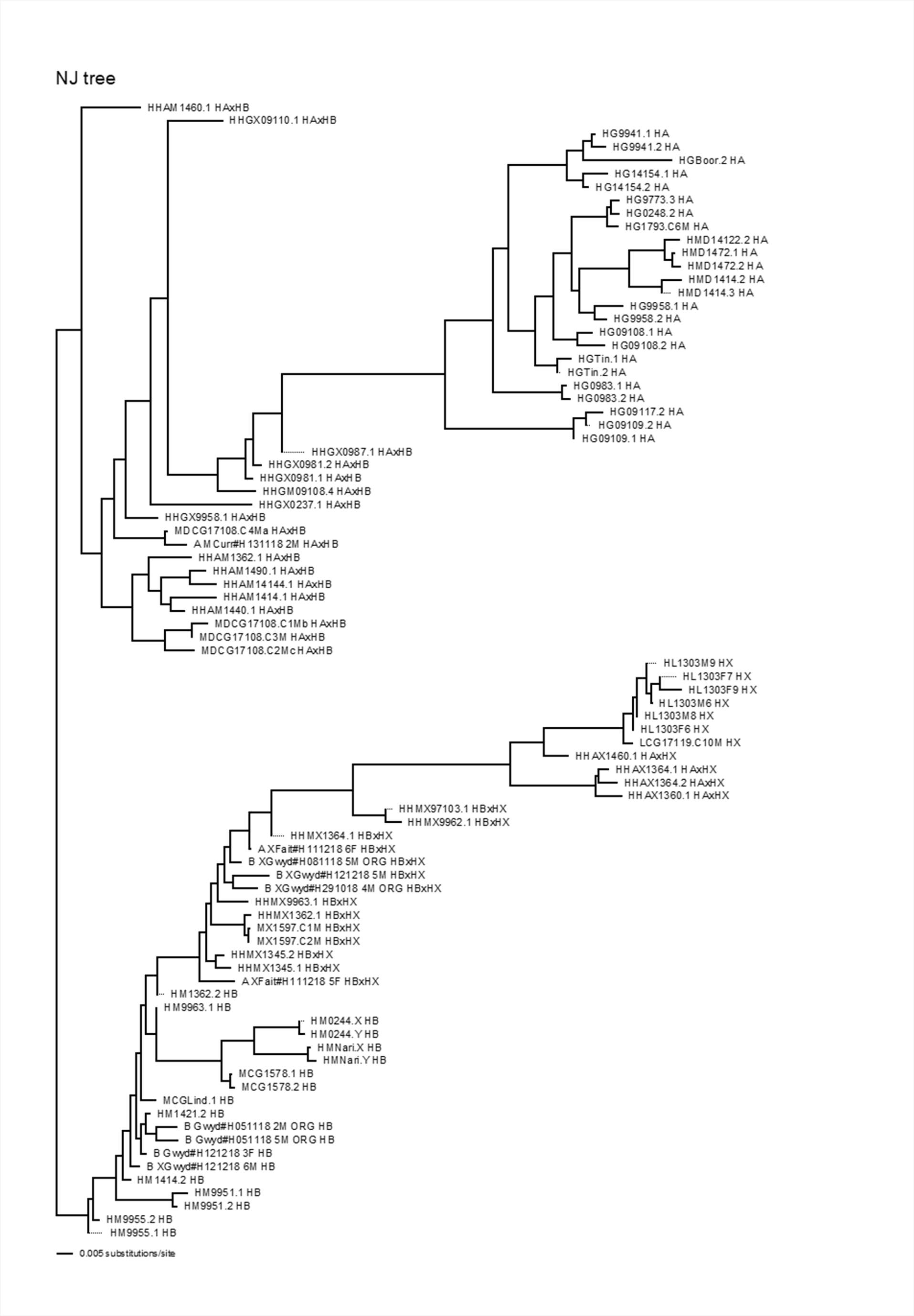

Supplement: evab030_Supplementary_Data [file evab030_supplementary_data.zip › Figure S2.tif]
